# Bidirectional and longitudinal associations among teacher–student relationships, peer relationships, and learning engagement in Chinese primary school students: a cross-lagged panel model

**DOI:** 10.3389/fpsyg.2025.1674600

**Published:** 2025-11-21

**Authors:** Jing Zhang, Chuanshi Liu

**Affiliations:** 1 School of Innovation and Entrepreneurship, Nanjing Vocational University of Industry Technology, Nanjing, China; 2 School of Psychology, Nanjing Normal University, Nanjing, China

**Keywords:** cross-lagged panel model, learning engagement, longitudinal study, peer relationships, primary school student, teacher–student relationships

## Abstract

**Introduction:**

In this study we aimed to investigate the longitudinal relationships among peer relationships, teacher–student relationships, and learning engagement in Chinese primary school students.

**Methods:**

This longitudinal study tracked 460 third-grade primary school students (mean age at T1 = 9.43 ± 0.68 years, 210 boys) through three waves of data collection (T1: June 2022, T2: January 2023, T3: October 2023) to assess their peer relationships, teacher–student relationships, and learning engagement.

**Results:**

The results revealed a persistent bidirectional relationship between peer relationships and learning engagement from grades 3 to 5. In contrast, the bidirectional relationship with teacher–student relationships was present only in grades 3 to 4 and disappeared by grade 5.

**Discussion:**

These results support the theory of developmental systems, suggesting that teacher-focused interventions in middle primary years and peer-mediated approaches in upper grades may optimize developmental outcomes.

## 1 Introduction

In recent years, the focus of educational research has progressively shifted from learning outcomes to learning processes (Ning and Yang, 2022). As a core indicator for assessing learning processes, learning engagement reflects individuals’ sustained positive attitudes and intrinsic motivation demonstrated during learning activities (Lam et al., 2014). Learning engagement constitutes a multidimensional construct, encompassing behavioral engagement (e.g., task completion, classroom interaction), cognitive engagement (e.g., critical thinking, strategy application), and affective engagement (e.g., interest and motivation) (Fredricks et al., 2004; Lam et al., 2014). Existing research posits that learning engagement not only significantly fosters student development by improving academic performance (Skinner and Raine, 2022) and reducing behavioral problems (Olivier et al., 2020) but also enhances subjective well-being (Zhu et al., 2019). The primary school years represent a critical developmental period characterized by high plasticity and have been shown to significantly predict future individual achievement (Chen et al., 2020). Thus, understanding how learning engagement develops during the primary school years holds significant value for educators.

The development of learning engagement is jointly influenced by multiple social–relational systems (Wang et al., 2019). Within the school context, teacher–student relationships and peer relationships represent the most influential forms of interpersonal interaction (Moreira and Lee, 2020). Current research suggests that learning engagement is a malleable state that can be influenced by school environments (Wang and Eccles, 2013) and exhibits significant associations with both peer relationships and teacher–student relationships (Yang et al., 2018; Zhen et al., 2021). Based on developmental systems theory (Gottlieb, 1991), individuals and environmental factors exist in constant dynamic interaction, mutually shaping one another. This perspective necessitates the adoption of longitudinal research designs to elucidate the bidirectional relationships between learning engagement and school-based interpersonal relationships. The theory emphasizes this reciprocal shaping across time, a dynamic that has received empirical validation in existing studies (Engels et al., 2016; Li et al., 2024). Furthermore, current findings are primarily derived from Western educational contexts, whereas the authoritative nature of teacher–student relationships and collectivist orientation in Chinese educational settings may yield distinct effects (Li et al., 2024). Therefore, examining the reciprocal relationship between Chinese primary school students’ school interpersonal relationships (teacher–student relationships and peer relationships) and learning engagement can enhance our understanding of the developmental mechanisms of engagement. To this end, this exploration investigates whether learning engagement exhibits culturally specific interaction patterns, thereby providing educators with targeted intervention strategies.

### 1.1 Peer relationships and learning engagement

Peer relationships, as a fundamental form of interpersonal dynamics in educational settings, refer to the social connections formed through interactions among individuals of similar ages or comparable psychological developmental levels (Zhou et al., 2015). Prior research has indicated that peer relationships play a significantly predictive role in students’ learning process (Engels et al., 2016). When interacting with peers, students experience a sense of acceptance and care. The feeling of belongingness within peer groups motivates students to engage more actively in learning activities (Wentzel et al., 2010). Grounded in self-determination theory (Ryan and Deci, 2020), the fulfillment of basic psychological needs fosters students’ sense of purpose and initiative, thereby enhancing their learning motivation and promoting deeper engagement in the learning process. Peer relationships provide a critical context for satisfying students’ fundamental psychological needs, including relatedness (Xuan et al., 2019), autonomy (De Loof et al., 2019), and competence (Bai and Gu, 2024).

Empirical evidence further confirms that positive peer relationships exert beneficial effects on students’ learning engagement (Shin and Chang, 2022; Zhou et al., 2024). For instance, empirical evidence indicates that peer friendships provide students with psychological and physical security, along with essential assistance in academic and social activities. These forms of emotional support and instrumental aid play a pivotal role in facilitating children’s more active engagement in classroom learning (Carmona-Halty et al., 2021). Another longitudinal study focusing on third and fourth graders demonstrated that negative peer relationships can predict academic performance declines over a 1-year period (Schwartz et al., 2005). Students can internalize effective learning strategies advocated for by their friends or acquire practical coping skills through observing positive peer role models (Bandura, 1986), which can contribute to their learning processes, such as learning engagement. Furthermore, when students collaborate with high-achieving peers on academic tasks, their learning processes may become particularly effective if these interactions prove more engaging and intellectually stimulating than those with average-achieving counterparts (Newcomb et al., 1993). Negative peer interactions, including aggressive behaviors, undermine student engagement by disrupting classroom climate and eliciting negative emotional responses (Furrer et al., 2014; Ladd et al., 2014).

Conversely, learning engagement may play a vital role in students’ peer relationships. Based on the peer-selection effect (Kandel, 1978), students demonstrate a distinct preference for forming relationships with peers who share similar characteristics. Previous research has consistently shown that students are more likely to establish friendships with academic peers at comparable learning levels (Brouwer et al., 2022). Consequently, learning engagement emerges as a significant factor influencing the formation of peer relationships. According to Mikami et al. (2017), learning engagement significantly contributes to positive peer relationships in late adolescence. Transactional models posit that individuals not only react to their interpersonal environment (i.e., students proactively enhance their learning engagement to adapt to surrounding contexts) but also actively shape it (i.e., students demonstrate a preference for forming relationships with highly engaged peers) (Sameroff, 2009). A growing body of empirical evidence supports the notion of a bidirectional relationship between peer relationships and learning engagement (Engels et al., 2016; Geng et al., 2019). Together, these findings underscore the reciprocal and dynamic nature of the connection between peer relationships and learning engagement.

Furthermore, existing research has predominantly drawn samples from secondary and tertiary education populations, with limited attention devoted to elementary school students (Berger et al., 2020; Mendoza and King, 2020). Thus, the current study aimed to add to this scarce knowledge base by examining transactional associations between peer relationships and learning engagement in primary school students.

### 1.2 Teacher–student relationships and learning engagement

Teacher–student relationships represent one of the most extensively studied social–relational constructs in educational environments, referring to meaningful emotional connections developed through sustained interactions between students and educators (Longobardi et al., 2016). Teacher–student relationships hold significant implications for student development, encompassing enhanced learning motivation (Chen et al., 2021), facilitated emotional intelligence development (Granero-Gallegos et al., 2023), and improved psychological well-being (Maricutoiu et al., 2023).

According to self-determination theory (Reeve, 2002; Ryan and Deci, 2020), when students experience autonomy-supportive contexts from authority figures (i.e., teachers and parents), their basic psychological needs for autonomy, competence, and relatedness are better satisfied. This need satisfaction fosters autonomous motivation and engagement, and can also contribute to a strengthened sense of competence and self-efficacy in learning. Students who maintain positive relationships with teachers gain access to broader support systems, including teacher encouragement (Sadoughi and Hejazi, 2022), constructive feedback (Tan et al., 2022), individualized attention (Kasperski and Blau, 2023), and supplementary academic assistance (Holzberger et al., 2019). These forms of support foster students’ feelings of being understood and valued, thereby enhancing their motivation to engage in learning (Lee et al., 2023). A consensus has emerged among researchers that positive teacher–student relationships serve as significant predictors of enhanced learning engagement in primary school students (Roorda et al., 2017; Zhen et al., 2021). For instance, a study on underachieving students found that positive teacher–student relationships can buffer the negative impact of peer rejection, thereby collectively improving classroom engagement (Li et al., 2024). This protective role is complemented by longitudinal evidence from Hasty et al. (2023), which indicates that such relationships also foster engagement indirectly by reducing externalizing behaviors. Further reinforcing these findings, Quin (2017) meta-analytic review established a significant, positive, and bidirectional relationship between teacher–student relationships and student engagement, suggesting that the two constructs mutually enhance each other over time.

Even as the fundamental bidirectional relationship gains empirical confirmation, the complexity of educational practice compels us to address more nuanced questions—that is, under what conditions, through which pathways, for which populations, at what developmental stages, and through what mechanisms these reciprocal effects operate. Therefore, the second objective of this study is to elucidate the association between teacher–student relationships and learning engagement among Chinese elementary students, while systematically investigating the nature of this bidirectional dynamic.

### 1.3 The current study

**Figure 1:**
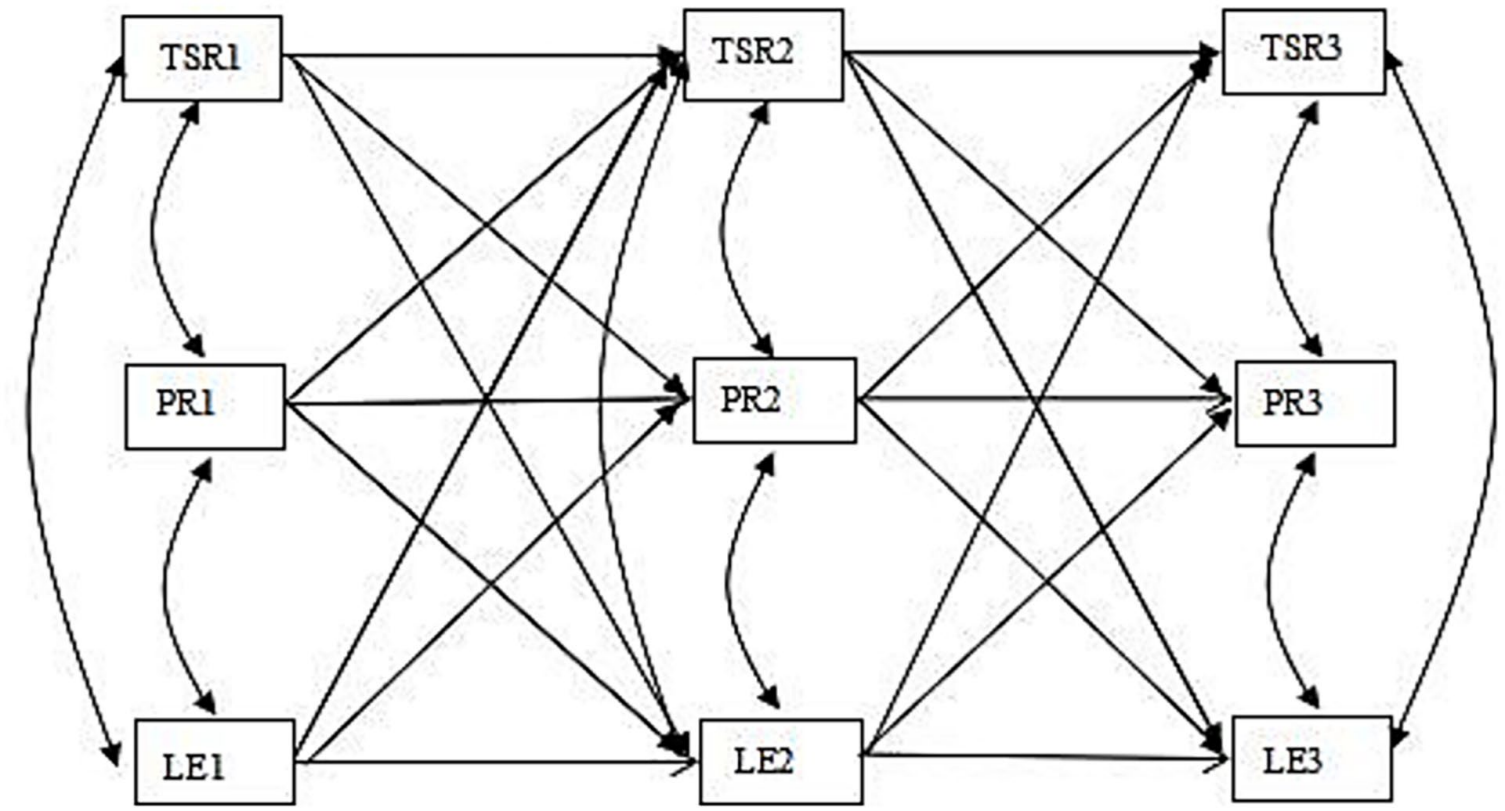
Cross-lagged associations among learning engagement (LE), teacher–student relationships (TSR), and peer relationships (PR), from time 1 (T1) to time 3 (T3). Gender and age are covariates.

The present study aimed to investigate the bidirectional relationship among teacher–student relationships, peer relationships, and learning engagement in Chinese primary school students across three time waves. Several hypotheses were tested to examine (1) whether there are reciprocal associations between peer relationships and learning engagement in primary school students and (2) whether there are reciprocal associations between teacher–student relationships and learning engagement in primary school students (Figure 1). By elucidating their bidirectional relationship, we can develop targeted interventions to enhance learning engagement among academically at-risk populations. Prior research has indicated that children’s age and gender are significantly correlated with their learning engagement and relationships (Hoffman et al., 2023; Xu et al., 2024). Therefore, this research will treat them as control variables.

Additionally, building on the recognized interconnectedness between these two social domains, we additionally examined bidirectional cross-lagged pathways between teacher–student relationships and peer relationships. These two social domains (teacher–student relationships and peer relationships) do not exist in isolation but are dynamically interconnected within the classroom ecosystem (Endedijk et al., 2022). A growing body of research demonstrates that teacher–student relationships can substantially shape the quality of students’ peer relationships (Bouchard and Smith, 2017; Hymel et al., 2015). In a recent meta-analysis, Endedijk et al. (2022) concluded that teachers play a pivotal role in influencing classroom peer dynamics. Specifically, by organizing collaborative activities, modeling respectful interactions, and managing classroom climate, teachers can directly and indirectly predict the development of peer relationships, which subsequently influences students’ social status and the quality of their peer interactions (Bierman, 2011). Peer relationships, as a central component of students’ social–emotional development, are closely linked to their learning engagement (Zhou et al., 2024).

Informed by theoretical frameworks emphasizing teachers’ contributions to peer ecology (Endedijk et al., 2022), we hypothesized that teacher–student relationships at T1 would indirectly influence learning engagement at T3 through their association with peer relationships at T2.

## 2 Methods

### 2.1 Participants

Participants included 460 Chinese grade 3 students from three primary schools (210 boys, mean age = 9.43 ± 0.679 years old). They were recruited from three public primary schools in Shandong province. We surveyed the students three times: in June 2022 (T1, *N* = 460, 210 boys), January 2023 (T2, *N* = 390, 173 boys), and June 2023 (T3, *N* = 328, 148 boys). Due to reasons such as student transfer or withdrawal during the second and third assessments, 70 and 62 participants were lost, respectively, resulting in a longitudinal attrition rate of 29% from T1 to T3. The analysis of participant attrition showed that there were no significant differences in learning engagement [*t*(457) = 1.02, *p* = 0.28], teacher–student relationships [*t*(447) = 1.30, *p* = 0.71], peer relationships [*t* (458) = 1.00, *p* = 0.67], and gender distribution [*χ*^2^(1) = 1.02, *p* = 0.31] between the dropouts and those who participated in the test three times at T1. All children spoke Chinese as their first language, and children with sensory deficits were excluded from the study. We also informed the students that their responses to the questionnaire were confidential and that we would only use the aggregated results. Parental consent was obtained prior to testing. This study was approved by the research ethics committee of the university.

### 2.2 Measures

#### 2.2.1 Peer relationships

The Peer Relationships Scale (Wang, 2013) is a 22-item scale designed to assess students’ peer relationships. Exemplary items include “I am concerned about how my classmates perceive me” and “I believe my classmates are playing a joke on me.” Students were asked to complete the 4-point Likert scale, ranging from “1 = strongly disagree” to “4 = strongly agree.” Higher scores indicate closer peer relationships (e.g., greater trust, support, and interaction frequency). The Cronbach’s alpha values from T1 to T3 were 0.89, 0.88, and 0.90, respectively. Confirmatory factor analysis revealed that the single-factor model demonstrates a good fit with the data (T1: *χ*^2^/df = 1.78, CFI = 0.99, TLI = 0.98, SRMR = 0.02, RMSEA = 0.04; T2: *χ*^2^/df = 1.85, CFI = 0.99, TLI = 0.98, SRMR = 0.02, RMSEA = 0.05; T3: *χ*^2^/df = 1.95, CFI = 0.98, TLI = 0.98, SRMR = 0.03, RMSEA = 0.05).

#### 2.2.2 Teacher–student relationships

To measure the interpersonal relationship between teachers and students, students were given the Teacher–Student Relationships Scale (Zou et al., 2007) and asked to complete it. This measure is a 22-item questionnaire (e.g., “My relationship with the teacher is intimate and warm” and “After class, I can freely say what I want to say to the teacher”) where students rate their relationships with teachers on a 5-point scale, from “1 = definitely does not apply” to “5 = definitely does apply.” The mean score of all items was calculated, with higher scores indicating stronger teacher–student relationships. The Cronbach’s alpha values from T1 to T3 were 0.89, 0.88, and 0.90, respectively. The confirmatory factor analysis for teacher–student relationships showed that the single-factor model fits well (T1: *χ*^2^/df = 2.99, CFI = 0.94, TLI = 0.93, SRMR = 0.03, RMSEA = 0.06; T2: *χ*^2^/df = 2.10, CFI = 0.91, TLI = 0.90, SRMR = 0.05, RMSEA = 0.06; T3: *χ*^2^/df = 2.39, CFI = 0.91, TLI = 0.90, SRMR = 0.06, RMSEA = 0.06).

#### 2.2.3 Learning engagement

The Learning Engagement Scale (Lam et al., 2014) is a 10-item scale that is composed of three dimensions: vitality (e.g., “when studying, I feel full of energy”), concentration (e.g., “learning inspires me”), and dedication (e.g., “I feel time passing quickly when studying”). Students were asked to complete these items on a 5-point scale ranging from “1 = completely disagree to 5 = completely agree.” The mean score of all items was calculated, with higher scores indicating greater levels of students’ learning engagement. The Cronbach’s alpha values from T1 to T3 were 0.86, 0.88, and 0.90, respectively. The confirmatory factor analysis for learning engagement showed that the models fit well (T1: *χ*^2^/df = 2.02, CFI = 0.92, TLI = 0.91, SRMR = 0.05, RMSEA = 0.07; T2: *χ*^2^/df = 2.18, CFI = 0.92, TLI = 0.91, SRMR = 0.07, RMSEA = 0.06; T3: *χ*^2^/df = 2.62, CFI = 0.91, TLI = 0.90, SRMR = 0.06, RMSEA = 0.05).

### 2.3 Procedure

Following the time-lagged design, the questionnaires were completed at three time points, including June 2022 (T1), January 2023 (T2), and June 2023 (T3). At each time point, children were group-tested in quiet classrooms supervised by two psychology graduate or undergraduate students who had received systematic training. At the commencement of each survey session, researchers delivered standardized instructions to guide students in independently completing the questionnaires. Upon completion, participants received small tokens of appreciation, and all questionnaires were collected for data processing. The duration of each survey was about 30–40 min. The order of measures was balanced across groups.

### 2.4 Data analyses

Missing data of learning engagement were.6 (T1), 11.3% (T2), and 11.9% (T3); missing data of peer relationships were 0 (T1), 9.8% (T2), and 11.9% (T3); and missing data of teacher–student relationships were 0 (T1), 11% (T2), and 11.9% (T3). The main reason for missing data was children’s omission of information when filling out the questionnaires. Missing values were missing in completely random fashion (Little’s MCAR test: χ^2^ = 55.28, df = 43, *p* = 0.10). Statistical analyses were performed using SPSS 22.0 (IBM Corporation, Armonk, NY, United States) and Mplus version 8.3 (Muthén and Muthén, Los Angeles, CA, United States). All models were estimated using Mplus version 8.3, with full-information maximum likelihood used to address missing data. First, a series of descriptive statistics and bivariate correlations were conducted. Second, the present study built a cross-lagged model to examine the relationships among teacher–student relationships, peer relationships, and learning engagement. Age and gender were controlled for in the models. In our data, males are encoded as 1 and females are encoded as 0. Model fit was assessed using multiple indices with contemporary thresholds (Putnick and Bornstein, 2016): CFI ≥ 0.95, TLI ≥ 0.95, RMSEA ≤0.06 (90% CI ≤ 0.08), and SRMR ≤0.08. Following recommendations by Marsh et al. (2004), we prioritized the joint criteria of CFI/TLI > 0.90 and RMSEA <0.08 for adequate fit, given the complexity of cross-lagged models. Measurement invariance tests confirmed scalar invariance for all constructs. For learning engagement, ΔCFI(T1–T3) = 0.006, ΔRMSEA = 0.002, indicating that the factor structure, loadings, and intercepts were equivalent over time. Similar results held for peer relationships (ΔCFI = 0.002, ΔRMSEA = 0.001) and teacher–student relationships (ΔCFI = 0.007, ΔRMSEA = 0.002).

## 3 Results

### 3.1 Common method bias

Common method bias can influence correlations among variables in self-reported studies (Tang and Wen, 2020). Additionally, we employed Harman’s single-factor test to test common method variance. Specifically, we set the common factor of all variables to 1, and each item of all variables was used as the explicit variable for confirmatory factorial analysis. The CFA showed that *χ*^2^/df = 7.02, CFI = 0.34, TLI = 0.32, SRMR = 0.22, and RMSEA = 0.22. The model fit was unsatisfactory, indicating that there was no common method bias.

### 3.2 Descriptive and correlational statistics

Table 1 displayed descriptive statistics and Pearson correlations among teacher–student relationships, peer relationships, and learning engagement at T1, T2, and T3. All variables correlated significantly (*p* < 0.05) across T1 to T3. The correlation coefficients for each variable ranged from.21 to.60, indicating medium positive correlations. Additionally, demographic variables such as gender and age were related to certain test outcomes.

**Table 1:** Correlation analysis results of teacher–student relationships, peer relationships, learning engagement, and control variables.

Variables	*M*	*SD*	Skew	Kurt	1.	2.	3.	4.	5.	6.	7.	8.	9.	10.	11.
1. Age1	9.43	0.68			1										
2. Gender					−0.02	1									
3. TSR1	3.79	0.71	−0.17	−0.59	0.12^*^	0.17^**^	1								
4. PR1	3.14	0.49	−0.11	−0.50	0.13^*^	0.10	0.58^**^	1							
5. LE1	3.37	0.89	−0.22	−0.71	0.11	0.11	0.47^**^	0.37^**^	1						
6. TSR2	3.79	0.67	−0.24	−0.72	0.07	0.12^*^	0.45^**^	0.35^**^	0.37^**^	1					
7. PR2	3.13	0.47	−0.16	−0.13	0.00	0.04	0.24^**^	0.46^**^	0.29^**^	0.59^**^	1				
8. LE2	3.58	1.00	−0.16	0.21	0.10	0.05	0.32^**^	0.33^**^	0.46^**^	0.60^**^	0.39^**^	1			
9. TSR3	3.71	0.66	−0.06	−0.58	0.12^*^	0.12^*^	0.41^**^	0.28^**^	0.30^**^	0.54^**^	0.25^**^	0.39^**^	1		
10. PR3	3.05	0.48	−0.41	−0.21	−0.02	−0.02	0.21^**^	0.31^**^	0.22^**^	0.31^**^	0.41^**^	0.32^**^	0.58^**^	1	
11. LE3	3.35	1.02	−0.25	−0.64	0.05	−0.08	0.29^**^	0.27^**^	0.44^**^	0.48^**^	0.35^**^	0.55^**^	0.52^**^	0.41^**^	1

TSR1, teacher–student relationships at T1; PR1, peer relationships at T1; LE1, learning engagement at T3; TSR2, teacher–student relationships at T2; PR2, peer relationships at T2; LE2, learning engagement at T2; TSR3, Teacher–student relationships at T3; PR3, peer relationships at T3; LE3, learning engagement at T3. *N* = 328. ^*^p < 0.05, ^**^p < 0.01, ^***^p < 0.001.

### 3.3 Cross-lagged path analysis

**Figure 2:**
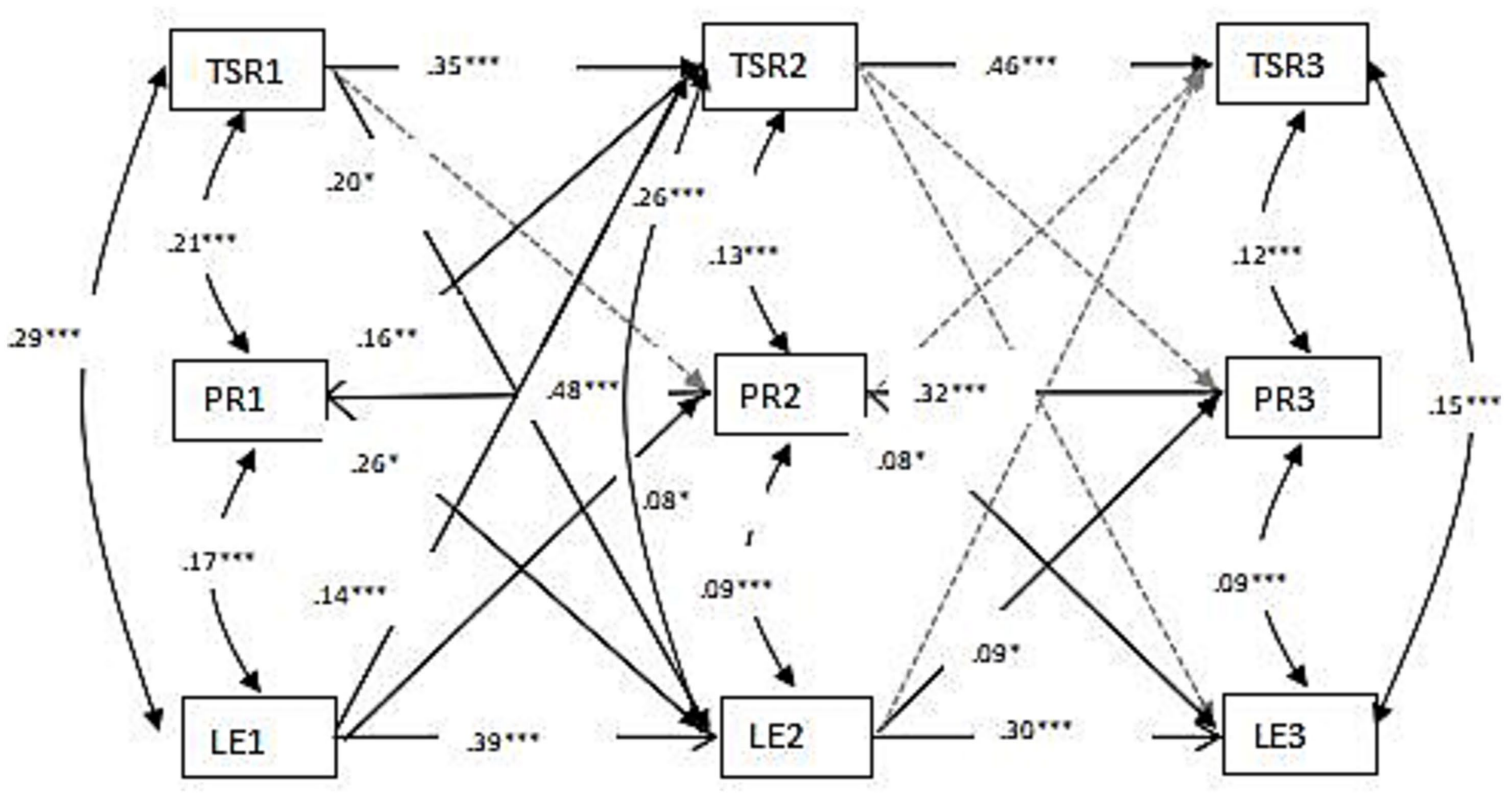
Cross-lagged associations among learning engagement (LE), teacher–student relationships (TSR), and peer relationships (PR), from time 1 (T1) to time 3 (T3). T1 = Grade 3, T2 = Grade 4, T3 = Grade 5. *p < 0.05, **p < 0.01, ***p < 0.001.

After controlling for gender and age, Figure 2 shows the parameter estimates and model fit statistics for the cross-lagged panel model, which demonstrated a good fit to the data, i.e., *χ*^2^(12) = 12.36, *p* = 0.42, CFI = 1.00, TLI = 0.99, RMSEA = 0.00, SRMR = 0.037. The autoregressive paths (Path TSR1 → TSR2 = 0.35, *p* < 0.001; Path TSR2 → TSR3 = 0.46, *p* < 0.001; Path PR1 → PR2 = 0.48, *p* < 0.001; Path PR2 → PR3 = 0.32, *p* < 0.001; Path LE1 → LE2 = 0.39, *p* < 0.001; Path LE2 → LE3 = 0.30, *p* < 0.001) indicate moderate-to-high stability, while the baseline covariances (Cov[TSR1, PR1] = 0.21, *p* < 0.001; Cov[LE1, PR1] = 0.17, *p* = <0.001; Cov[TSR1, LE1] = 0.29, *p* < 0.001) account for initial associations between variables.

Learning engagement at each time point (Times 1–3) was significantly associated with peer relationships (T1: *r* = 0.17, *p* < 0.001; T2: *r* = 0.09, *p* < 0.001; T3: *r* = 0.09, *p* < 0.001) and teacher–student relationships (T1: *r* = 0.29, *p* < 0.001; T2: *r* = 0.26, *p* < 0.001; T3: *r* = 0.15, *p* < 0.001) at the corresponding time point. Moreover, teacher–student relationships at T1 significantly predicted learning engagement at T2 (*β* = 0.20, SE = 0.08, *p* < 0.001), and learning engagement at T1 accounted for teacher–student relationships at T2 (*β* = 0.14, SE = 0.04, *p* < 0.001). However, the cross-lagged paths between teacher–student relationships and learning engagement from T2 to T3 were non-significant. In addition, peer relationships at T1 significantly predicted learning engagement at T2 (*β* = 0.26, SE = 0.12, *p* < 0.05), learning engagement at T1 accounted for peer relationships at T2 (*β* = 0.08, SE = 0.03, *p* < 0.05), peer relationships at T2 significantly predicted learning engagement at T3 (*β* = 0.08, SE = 0.06, *p* < 0.05), and learning engagement at T2 accounted for peer relationships at T3 (*β* = 0.09, SE = 0.03, *p* < 0.05). Contrary to the expectation of a reciprocal relationship, the cross-lagged effects were not symmetrical between teacher–student relationships and peer relationships. The only significant path found was from earlier peer relationships to later teacher–student relationships (PR1 → TSR2). Since the path in the opposite direction (from teacher–student relationships to peer relationships) was non-significant, the results do not provide evidence for a bidirectional interplay over time. All paths from the covariates to peer relationships, teacher–student relationships, and learning engagement were non-significant (standardized coefficients ranged from −0.05 to.02, all *p* > 0.10).

In brief, our data suggest there exists a mutual relationship between peer relationships and learning engagement, whereas the mutual relationship between teacher–student relationships and learning engagement seems to be unstable. No bidirectional relationship was observed between teacher–student relationships and peer relationships.

## 4 Discussion

This study examined the bidirectional relationships among peer relationships, teacher–student relationships, and learning engagement in Chinese primary school students. The results demonstrated that children’s peer relationships consistently predicted learning engagement, indicating that peer relationships at prior time points significantly predicted subsequent learning engagement. However, the bidirectional relationship between teacher–student relationships and learning engagement was not stable. This result supports the view of the developmental systems theory (Gottlieb, 1991) that child development is a process of continuous interaction between social relationships and individual behavior. The current study extends existing literature by revealing the bidirectional association between peer relationships and learning engagement among Chinese children, providing empirical evidence for the connection between classroom interpersonal dynamics and learning engagement development.

### 4.1 Bidirectional relationship between peer relationships and learning engagement

This study showed that peer relationships were concurrently and positively related to learning engagement at each time point, in line with previous research (Yang et al., 2024). In addition, the current results indicated a long-term and dynamic bidirectional relationship between peer relationships and learning engagement. Consistent with prior research, interpersonal relationships between individuals and friends, peers, and classmates can predict changes in individual engagement throughout the academic year (Fredricks et al., 2018). The highly stable autoregressive paths observed in this study demonstrate strong temporal stability in students’ teacher–student relationships, peer relationships, and learning engagement, which renders the significant cross-lagged effects we detected particularly noteworthy.

This finding aligns with self-determination theory (Ryan and Deci, 2020), positing that high-quality peer relationships fulfill students’ basic psychological needs and thereby facilitate their learning engagement. Specifically, positive peer relationships characterized by egalitarian communication and non-controlling interactions can mitigate external pressures, prompting children to engage in learning behaviors autonomously rather than through coercion (De Laet et al., 2015). Primary school students develop a sense of belonging through peer friendships and obtain essential support in academic and social activities (Collie, 2022; Li and Li, 2021), and these emotional and instrumental forms of assistance play a vital role in promoting their classroom learning engagement (Hakimzadeh et al., 2016). Peer involvement, serving as instrumental support, helps students track and complete academic tasks, which satisfies their need for competence and thereby elevates their effort in learning tasks, ultimately enhancing their engagement (Bai and Gu, 2024).

Furthermore, learning engagement also contributed to peer relationships from grades 3–5, consistent with previous research findings (Engels et al., 2016; Li et al., 2024). Multiple potential explanations exist for this relationship across dimensions of learning engagement, such as emotional, behavioral, and cognitive engagement (Chang et al., 2016). First, from the perspective of emotional engagement, Kindermann (1993) observed that, when learners are highly engaged, their behaviors demonstrate initiative and are accompanied by positive emotions such as enjoyment and interest. When students communicate about learning experiences and share academic enjoyment with peers, such interactions may stimulate mutual interest in learning. Shared academic interests can serve as significant bonds among peers, facilitating interpersonal communication (Pan, 2017) and thereby deepening their relationships. Second, regarding behavioral engagement, children with high learning involvement demonstrate positive behaviors such as concentration, diligence, and effort during learning activities (Lei et al., 2024). Such positive behavioral patterns earn admiration and favor from peers, thereby fostering positive peer relationships (Fletcher, 2012). During collective activities, children gain increased opportunities for mutual observation and competitive motivation (Keller and Lowenstein, 2011), which enhances behavioral efficiency. When children observe peers’ learning engagement and progress, it often triggers competitive awareness and self-improvement efforts. Such constructive competition facilitates mutual encouragement among peers (Olaya and González-González, 2020), consequently contributing to the establishment of positive peer relationships. Third, from the perspective of cognitive engagement, children with high learning proactivity accumulate greater knowledge and skills during the learning process (Harbour et al., 2015). They actively share these cognitive resources with peers and assist in resolving academic challenges (Crowley-Cyr and Hevers, 2021). Such knowledge sharing not only enhances peers’ academic performance but also strengthens mutual trust and friendship (Warneken, 2018). Prosocial and reciprocal behaviors serve as crucial mechanisms for establishing peer relationships, including friendships (Bagwell and Schmidt, 1989). Children who actively engage in learning feel valued when receiving assistance, thereby reinforcing positive peer relationships.

### 4.2 Bidirectional relationship between learning engagement and teacher–student relationships

The results showed that the teacher–student relationship predicted learning engagement from T1 to T2, consistent with previous studies (Yang et al., 2024; Zhen et al., 2021). Our results support developmental systems theory (Gottlieb, 1991), which posits that positive teacher–student relationships foster students’ engagement in learning, which in turn promotes more proactive and profound pedagogical interactions. This reciprocal dynamic drives their synergistic evolution through mutual enforcement. Specifically, students with high learning engagement hope to achieve academic success, which is consistent with teachers’ educational goals. Having this common goal will promote the formation of cooperative relationships between teachers and students, wherein communication and exchange between teachers and students increase (Liu et al., 2017), mutual understanding deepens, and relationships become more harmonious. Also, in the process of learning engagement, there will be more emotional communication between teachers and students (Hart et al., 2011). Students may seek help and support from teachers due to learning difficulties, and teachers will also care about students’ learning and living conditions (Birch and Ladd, 1997). This kind of emotional communication helps establish lasting connections between teachers and students (Algoe et al., 2013). The fulfillment of students’ relational needs subsequently increases their willingness to invest effort in learning (Reeve, 2002). Furthermore, when students are deeply engaged in their studies, they actively participate in class discussions, ask questions, and diligently complete assignments (Reyes et al., 2012). Students’ proactive engagement in learning allows teachers to appreciate their passion and dedication and fosters positive evaluations, highlighting their drive for self-improvement and curiosity (Chen et al., 2021). Teachers will feel that interacting with such students is more meaningful, thereby enhancing their goodwill and attention toward them and promoting the healthy development of teacher–student relationships. In summary, teachers often give more attention and encouragement to students with a positive learning attitude; at the same time, teacher feedback can also help students understand their learning progress and shortcomings, promoting them to further increase their learning engagement (Martin and Rimm-Kaufman, 2015). In such ways, teacher–student relationships and learning engagement will enter into a virtuous cycle.

However, the bidirectional relationship disappears from T2 to T3 time points, and there is a stable bidirectional relationship between peer relationships and learning engagement during T1–T3. This contradicts previous research results (Vollet and Kindermann, 2020). The current research conclusion implies that, in early stages, teacher–student relationships significantly predicted children’s learning engagement. However, in the upper grades of primary school, teacher–student relationships may not be the primary reason influencing learning engagement. There are several reasons for this. Hartup (1989) distinguished the interpersonal relationships children have with one another into vertical relationships (like those with teachers) and horizontal relationships (like those with peers). During the socialization process, horizontal relationships exert a more profound and extensive influence on children than vertical ones. For young children, both teacher–student and peer relationships are equally significant. On the one side, as children age, peer relationships may assume even greater importance (Newcomb et al., 1993). Other variables concerning teachers primarily influence primary school students’ learning engagement, such as teacher style (Ahlfeldt et al., 2005), teacher support (Hughes and Cao, 2018), and teaching strategies (Henrie et al., 2015). On the other hand, our findings align with established socio-emotional developmental trajectories (Chen and Wu, 2022; Roorda et al., 2017). In early primary years, teachers function as pivotal authority figures and primary sources of security, exerting profound influence on student development (García-Rodríguez et al., 2023). As students progress into upper grades and undergo the transition toward adolescence, their pursuit of autonomy intensifies, while their social focus decisively shifts toward peer groups (Sidanius and Pratto, 1999; Ye and Pang, 1999). Consequently, although teacher–student relationships maintain importance, their longitudinal predictive power over learning engagement and peer relationships demonstrates relative attenuation, reflecting the dynamic restructuring of individuals’ social ecosystems throughout development. Previous research has demonstrated that teacher–student relationships among third to sixth graders tend to weaken as students advance through grades, with cognitive maturation and increased academic demands gradually attenuating emotional connections between teachers and students (Chen and Wu, 2022). Another empirical study has shown that, in grades 1–3 of primary school, the impact of teacher–student relationships on academic engagement is significantly greater than that of peer relationships, while, in grades 4–6 of primary school, the effect of peer relationships exceeds that of teacher–student relationships (Roorda et al., 2017). Furthermore, in terms of curriculum reform, there have been phased changes in the difficulty and structure of the curriculum for grades 4–5 in Chinese primary schools (Liu et al., 2024), which may accelerate the need for peer assistance. Therefore, when children are in the upper grades of primary school, the teacher–student relationship cannot predict learning engagement.

Inconsistent with theoretical expectations (Endedijk et al., 2022), the path from T1 teacher–student relationships to T2 peer relationships was not significant (*β* = 0.00, *p* = 0.97). Consequently, the analysis did not provide support for the hypothesized mediating role of peer relationships in the link between teacher–student relationships and subsequent learning engagement. This suggests that, within our specific research context and sample, the predictive effect of teacher–student relationships on subsequent learning engagement may not be primarily explained through the mechanism of enhancing peer relationships. Future research involving diverse age groups, cultural contexts, or increased measurement occasions is warranted to clarify the boundary conditions under which this mediating pathway may emerge. In addition, the analytical results indicated that the paths from covariates (gender, age) to the primary variables (peer relationships, teacher–student relationships, and learning engagement) all failed to reach statistical significance, demonstrating that the cross-lagged relationships among peer relationships, teacher–student relationships, and learning engagement are robust and unlikely to be confounded by these demographic factors.

### 4.3 Limitations and future work

Our research provides new and longitudinal evidence about the relationship between school interpersonal relationships and learning engagement. However, this study has some limitations. First, one notable limitation of this study is the unequal time intervals between our data-collection points. This design feature prevented us from imposing and testing equality constraints on the autoregressive and cross-lagged paths over time (Zyphur et al., 2021). Consequently, we could not rigorously examine whether the strengths of the dynamic processes between variables remained stable or changed across different developmental periods. Future research can employ longitudinal designs with equally spaced measurement waves. Such a design would enable direct tests of temporal invariance, thereby providing deeper insights into the stability and evolution of teacher–student relationships, peer relationships, and learning engagement over time. Second, our model focused exclusively on school-based factors (teacher–student and peer relationships). We did not include family-related variables, such as parenting styles or the home learning environment, which are also known to significantly shape children’s learning engagement (Yang et al., 2024). Future research should adopt a more comprehensive ecological framework by integrating both school and family factors to disentangle their unique and interactive effects on student learning engagement. Third, it is equally important to acknowledge the limitations of the approaches employed to assess common method variance. Future research could employ more robust statistical techniques, such as the unmeasured latent method factor test or the marker variable technique, to provide a more definitive evaluation of potential common method bias (Tang and Wen, 2020). Fourth, our cross-lagged panel model used observed variables, which precludes the control of measurement error. Future research would benefit from employing latent variable–modeling techniques, such as the random intercept cross-lagged panel model (Hamaker et al., 2015). This advanced approach would allow for a more precise disentanglement of stable between-person differences from dynamic within-person processes, thereby providing a clearer interpretation of the temporal dynamics between teacher–student relationships, peer relationships, and learning engagement. Finally, regarding methodology, future work should move beyond sole reliance on self-reports to measure learning engagement. Adopting a multi-method, multi-informant approach—complementing self-reports by integrating classroom observations, interviews, and reports from teachers and peers—would yield more comprehensive and robust data (Kim and Cho, 2025).

## 5 Conclusion and implications

Our findings showed bidirectional relations between peer relationships and learning engagement in Chinese students from grades 3–5. Furthermore, there is a bidirectional relationship between teacher–student relationships and learning engagement in primary school grades 3 and 4, which disappeared from grades 4–5.

To a certain extent, this once again confirms the important impact of peer relationships on children’s learning engagement, and this impact is not only immediate and static but can also have a long-term effect on the dynamic development of children’s learning engagement over time. This offers guidance for precisely targeting interventions in students’ learning engagement. Consequently, it may be necessary to prioritize attention toward student cohorts exhibiting suboptimal peer relationships, who may be experiencing certain levels of socio-emotional adaptation difficulties. Educators can use simple sociometric tools or observational checklists to identify socially isolated students at an early stage, enabling timely interventions to facilitate their integration into peer networks. In instructional design, integrating social–emotional learning into daily academic teaching through cooperative learning structures is recommended (Cipriano et al., 2023). This pedagogical approach not only supports knowledge acquisition but also explicitly fosters positive peer interactions and collaborative skills (Lyons et al., 2021). Beyond methodology, teachers should develop warm, supportive, and responsive relationships that extend past academic concerns, deliberately cultivating students’ sense of trust and belonging (Qi et al., 2024).

## Data Availability

The raw data supporting the conclusions of this article will be made available by the authors, without undue reservation.

## References

[ref1] Ahlfeldt S. Mehta S. Sellnow T. (2005). Measurement & analysis of student engagement in university classes where varying levels of PBL methods of instruction are in use. High. Educ. Res. Dev. 24, 5–20. doi: 10.1080/0729436052000318541, PMID: 41221155

[ref2] Algoe S. B. Fredrickson B. L. Gable S. L. (2013). The social functions of the emotion of gratitude via expression. Emotion 13, 605–609. doi: 10.1037/a0032701, PMID: 23731434

[ref3] Bagwell C. L. Schmidt M. E. (1989). Friendships in childhood and adolescence, vol. 63. New York, NY, USA: Guilford Publications, 377–416.

[ref4] Bai X. Gu X. (2024). Contribution of self-determining theory to k-12 students' online learning engagements: research on the relationship among teacher support dimensions, students' basic psychological needs satisfaction, & online learning engagements. Educ. Technol. Res. Dev. 72, 2939–2961. doi: 10.1007/s11423-024-10383-9

[ref5] Bandura A. (1986). Social foundations of thought and action: a social cognitive theory. Englewood Cliffs, NJ: Prentice-Hall.

[ref7] Berger C. Deutsch N. Cuadros O. Franco E. Rojas M. Roux G. (2020). Adolescent peer processes in extracurricular activities: identifying developmental opportunities. Child Youth Serv. Rev. 118:105457. doi: 10.1016/j.childyouth.2020.105457

[ref8] Bierman K. L. (2011). The promise and potential of studying the “invisible hand” of teacher influence on peer relations and student outcomes: a commentary. J. Appl. Dev. Psychol. 32, 297–303. doi: 10.1016/j.appdev.2011.04.004

[ref9] Birch S. H. Ladd G. W. (1997). The teacher-student relationship and children's early school adjustment. J. Sch. Psychol. 35, 61–79. doi: 10.1016/S0022-4405(96)00029-5

[ref10] Bouchard K. L. Smith J. D. (2017). Teacher-student relationship quality & children’s bullying experiences with peers: reflecting on the mesosystem. Educ. Forum 81, 108–125. doi: 10.1080/00131725.2016.1243182

[ref12] Brouwer J. de Matos Fernandes C. Steglich C. Jansen E. Hofman W. H. Flache A. (2022). The development of peer networks and academic performance in learning communities in higher education. Learn. Instr. 80:101603. doi: 10.1016/j.learninstruc.2022.101603

[ref13] Carmona-Halty M. Salanova M. Llorens S. Schaufeli W. B. (2021). Linking positive emotions and academic performance: the mediated role of academic psychological capital and academic engagement. Curr. Psychol. 40, 2938–2947. doi: 10.1007/s12144-019-00227-8

[ref14] Chang D. F. Chien W. C. Chou W. C. (2016). Meta-analysis approach to detect the effect of student engagement on academic achievement. ICIC Express Lett. 10, 2241–2246.

[ref15] Chen P. Bao C. Gao Q. (2021). Proactive personality & academic engagement: the mediating effects of teacher-student relationships and academic self-efficacy. Front. Psychol. 12:652994. doi: 10.3389/fpsyg.2021.652994, PMID: 34168588 PMC8217667

[ref16] Chen J. Huebner E. S. Tian L. (2020). Longitudinal relations between hope and academic achievement in elementary school students: behavioral engagement as a mediator. Learn. Individ. Differ. 78:101824. doi: 10.1016/j.lindif.2020.101824

[ref17] Chen Y. Wu Z. (2022). The impact of perceived class atmosphere on learning engagement among special education teacher trainees: the mediating role of professional identity and the moderating role of future orientation. Psychol. Dev. Educ. 38, 244–253. doi: 10.16187/j.cnki.issn1001-4918.2022.02.11

[ref18] Cipriano C. Strambler M. Naples L. H. Ha C. Kirk M. A. Wood M. . (2023). The state of evidence for social and emotional learning: a contemporary meta-analysis of universal school-based SEL interventions. Child Dev. 94, 1181–1204. doi: 10.1111/cdev.13968, PMID: 37448158

[ref19] Collie R. J. (2022). Social-emotional need satisfaction & students’ academic engagement and social-emotional skills. Educ. Psychol. 44, 117–135. doi: 10.1080/01443410.2024.2324751, PMID: 41221155

[ref20] Crowley-Cyr L. Hevers J. (2021). Using peer assisted learning to improve academic engagement and progression of first year online law students. J. Univ. Teach. Learn. Pract. 18, 1–17. doi: 10.53761/1.18.1.2

[ref21] De Laet S. Colpin H. Vervoort E. Doumen S. Van Leeuwen K. Goossens L. . (2015). Developmental trajectories of children’s behavioral engagement in late elementary school: both teachers and peers matter. Dev. Psychol. 51, 1292–1306. doi: 10.1037/a0039478, PMID: 26192040

[ref22] De Loof H. Struyf A. Boeve-De Pauw J. Van Petegem P. (2019). Teachers' motivating style and students' motivation & engagement in stem: the relationship between three key educational concepts. Res. Sci. Educ. 51, 109–127. doi: 10.1007/s11165-019-9830-3, PMID: 41215982

[ref23] Endedijk H. M. Breeman L. D. Van Lissa C. J. Hendrickx M. M. H. G. Den Boer L. Mainhard T. (2022). The teacher's invisible hand: a meta-analysis of the relevance of teacher–student relationship quality for peer relationships and the contribution of student behavior. Rev. Educ. Res. 92, 370–412. doi: 10.3102/00346543211051428

[ref24] Engels M. C. Colpin H. van Leeuwen K. Bijttebier P. van den Noortgate W. Claes S. . (2016). Behavioral engagement, peer status, and teacher-student relationships in adolescence: a longitudinal study on reciprocal influences. J. Youth Adolesc. 45, 1192–1207. doi: 10.1007/s10964-016-0414-5, PMID: 26759132

[ref25] Fletcher J. M. (2012). Similarity in peer college preferences: new evidence from Texas. Soc. Sci. Res. 41, 321–330. doi: 10.1016/j.ssresearch.2011.11.001, PMID: 23017754

[ref26] Fredricks J. A. Blumenfeld P. C. Paris A. H. (2004). School engagement: potential of the concept, state of the evidence. Rev. Educ. Res. 74, 59–109. doi: 10.3102/00346543074001059

[ref26000] Fredricks J. A. Hofkens T. Wang M. T. Mortenson E. Scott P. (2018). Supporting girls’ and boys’ engagement in math and science learning: A mixed methods study. J. Res. Sci. Teach. 55, 271–298. doi: 10.1002/tea.21419

[ref27] Furrer C. J. Skinner E. A. Pitzer J. R. (2014). The influence of teacher & peer relationships on students’ classroom engagement and everyday resilience. Nat I. Soc. Stud. Educ. 116, 101–123. doi: 10.1177/016146811411601319, PMID: 41220966

[ref28] García-Rodríguez L. Redín C. Abaitua C. (2023). Teacher-student attachment relationship, variables associated, and measurement: a systematic review. Educ. Res. Rev. 38:100488. doi: 10.1016/j.edurev.2022.100488

[ref29] Geng L. Zheng Q. Zhong X. Li L. (2019). Longitudinal relations between students' engagement and their perceived relationships with teachers & peers in a Chinese secondary school. Asia-Pac. Educ. Res. 29, 171–181. doi: 10.1007/s40299-019-00463-3, PMID: 41215982

[ref30] Gottlieb G. (1991). Experiential canalization of behavioral development: results. Dev. Psychol. 27, 35–39. doi: 10.1037//0012-1649.27.1.35

[ref31] Granero-Gallegos A. Gómez-López M. Manzano-Sánchez D. (2023). Effect of a physical education teacher's autonomy support on self-esteem in secondary-school students: the mediating role of emotional intelligence. Children 10:1690. doi: 10.3390/children10101690, PMID: 37892354 PMC10605116

[ref32] Hakimzadeh R. Besharat M. A. Khaleghinezhad S. A. Ghorban Jahromi R. (2016). Peers' perceived support, student engagement in academic activities and life satisfaction: a structural equation modeling approach. Sch. Psychol. Int. 37, 240–254. doi: 10.1177/0143034316630020

[ref33] Hamaker E. L. Kuiper R. M. Grasman R. P. P. P. (2015). A critique of the cross-lagged panel model. Psychol. Methods 20, 102–116. doi: 10.1037/a0038889, PMID: 25822208

[ref34] Harbour K. E. Evanovich L. L. Sweigart C. A. Hughes L. E. (2015). A brief review of effective teaching practices that maximize student engagement. Pre. Sch. Fail. 59, 5–13. doi: 10.1080/1045988X.2014.919136

[ref35] Hart S. R. Stewart K. Jimerson S. R. (2011). The student engagement in schools questionnaire (SESQ) and the teacher engagement report form-new (TERF-N): examining the preliminary evidence. Contemp. Sch. Psychol. 15, 67–79. doi: 10.1007/BF03340964

[ref36] Hartup W. W. (1989). Social relationships and their developmental significance. Am. Psychol. 44, 120–126. doi: 10.1037/0003-066X.44.2.120

[ref37] Hasty L. M. Quintero M. Li T. Song S. Wang Z. (2023). The longitudinal associations among student externalizing behaviors, teacher-student relationships, and classroom engagement. J. Sch. Psychol. 100:101242. doi: 10.1016/j.jsp.2023.101242, PMID: 37689439

[ref38] Henrie C. R. Halverson L. R. Graham C. R. (2015). Measuring student engagement in technology-mediated learning:a review. Comput. Educ. 90, 36–53. doi: 10.1016/j.compedu.2015.09.005

[ref39] Hoffman A. Kurtz C. B. Dumas F. Loose F. Smeding A. Régner I. (2023). The development of gender stereotypes about academic aptitude among European French and north African French boys. Eur. J. Dev. Psychol. 20, 24–49. doi: 10.1080/17405629.2021.2012144

[ref40] Holzberger D. Praetorius A.-K. Seidel T. Kunter M. (2019). Identifying effective teachers: the relation between teaching profiles and students’ development in achievement and enjoyment. Eur. J. Dev. Psychol.Eur. J. Psychol. Educ. 34, 801–823. doi: 10.1007/s10212-018-00410-8

[ref41] Hughes J. N. Cao Q. (2018). Trajectories of teacher-student warmth and conflict at the transition to middle school: effects on academic engagement and achievement. J. Sch. Psychol. 67, 148–162. doi: 10.1016/j.jsp.2017.10.003, PMID: 29571530 PMC5868433

[ref42] Hymel S. Mcclure R. Miller M. Shumka E. Trach J. (2015). Addressing school bullying: insights from theories of group processes. J. Appl. Dev. Psychol. 37, 16–24. doi: 10.1016/j.appdev.2014.11.008

[ref43] Kandel D. B. (1978). Homophily, selection, and socialization in adolescent friendships. Am. J. Sociol. 84, 427–436. doi: 10.1086/226792

[ref44] Kasperski R. Blau I. (2023). Social capital in high-schools: teacher-student relationships within an online social network and their association with in-class interactions & learning. Interact. Learn. Environ. 31, 955–971. doi: 10.1080/10494820.2020.1815220

[ref45] Keller J. Loewenstein J. (2011). The cultural category of cooperation: a cultural consensus model analysis for China and the United States. Organ. Sci. 22, 299–319. doi: 10.1287/orsc.1100.0530, PMID: 19642375

[ref46] Kim J. Cho H. (2025). Which pathways do teachers experience before deciding to leave their schools? Exploring korean teachers' attribution to challenges faced while teaching migrant students. Teach. Teach. Educ. 156:104935. doi: 10.1016/j.tate.2025.104935

[ref47] Kindermann T. A. (1993). Natural peer groups as contexts forindividual development: the case of children's motivation in school. Dev. Psychol. 29, 970–977. doi: 10.1037/0012-1649.29.6.970

[ref48] Ladd G. W. Ettekal I. Kochenderfer-Ladd B. Rudolph K. D. Andrews R. K. (2014). Relations among chronic peer group rejection, maladaptive behavioral dispositions, and early adolescents' peer perceptions. Child Dev. 85, 971–988. doi: 10.1111/cdev.12214, PMID: 24397253

[ref49] Lam S. F. Jimerson S. Wong B. P. H. Kikas E. Zollneritsch J. (2014). Understanding and measuring student engagement in school: the results of an international study from 12 countries. Sch. Psychol. Q. 29, 213–232. doi: 10.1037/spq0000057, PMID: 24933218

[ref50] Lee T. Hong S. E. Kang J. Lee S. M. (2023). Role of achievement value, teachers' autonomy support, and teachers' academic pressure in promoting academic engagement among high school seniors. S*ch. Psychol. Int.* 44, 629–648. doi: 10.1177/01430343221150748, PMID: 41220966

[ref51] Lei H. Chen C. Luo L. (2024). The examination of the relationship between learning motivation and learning effectiveness: a mediation model of learning engagement. Curr. Psychol. 11:137. doi: 10.1057/s41599-024-02666-6, PMID: 39310270

[ref52] Li X. Li Y. (2021). Empirical research and analysis of foreign learning engagement based on systematic literature review. Res. Mod. Distance Educ. 33, 83–95. doi: 10.3969/j.issn.1009-5195.2021.02.008

[ref53] Li T. Wang Z. Merrin G. J. Wan S. Bi K. Quintero M. (2024). The joint operations of teacher-student and peer relationships on classroom engagement among low-achieving elementary students: a longitudinal multilevel study. Contem. Educ. Psychol. 77:102258. doi: 10.1016/j.cedpsych.2024.102258, PMID: 38463698 PMC10922620

[ref54] Liu H. Hou H. Yu W. Li M. (2024). Basic Education Reform & Discipline Construction (written discussion). Educat. Sci. 40, 1–14.

[ref55] Liu R.-D. Zhen R. Ding Y. Liu Y. Wang J. Jiang R. . (2017). Teacher support and math engagement: roles of academic self-efficacy and positive emotions. Educ. Psychol. 38, 3–16. doi: 10.1080/01443410.2017.1359238, PMID: 41221155

[ref56] Longobardi C. Prino L. E. Marengo D. Settanni M. (2016). Student−teacher relationships as a protective factor for school adjustment during the transition from middle to high school. Front. Psychol. 7:1988. doi: 10.3389/fpsyg.2016.01988, PMID: 28066305 PMC5179523

[ref57] Lyons K. M. Lobczowski N. G. Greene J. A. Whitley J. Mclaughlin J. E. (2021). Using a design-based research approach to develop and study a web-based tool to support collaborative learning. Comput. Educ. 161:104064. doi: 10.1016/j.compedu.2020.104064

[ref58] Maricutoiu L. P. Pap Z. Stefancu E. Mladenovici V. Valache D. G. Popescu B. D. . (2023). Is teachers' well-being associated with students' school experience? A meta-analysis of cross-sectional evidence. Educ. Psychol. Rev. 35:1. doi: 10.1007/s10648-023-09721-9

[ref59] Marsh H. W. Hau K. T. Wen Z. (2004). In search of golden rules: comment on hypothesis-testing approaches to setting cutoff values for fit indexes and dangers in overgeneralizing hu and Bentler’s (1999) findings. Struct. Equ. Modeling 11, 320–341. doi: 10.1207/s15328007sem1103_2

[ref60] Martin D. P. Rimm-Kaufman S. E. (2015). Do student self-efficacy and teacher-student interaction quality contribute to emotional & social engagement in fifth grade math? Sch. Psychol. 53, 359–373. doi: 10.1016/j.jsp.2015.07.001, PMID: 26407834

[ref61] Mendoza N. B. King R. B. (2020). The social contagion of student engagement in school. Sch. Psychol. Int. 41, 454–474. doi: 10.1177/0143034320946803

[ref62] Mikami A. Y. Ruzek E. A. Hafen C. A. Gregory A. Allen J. P. (2017). Perceptions of relatedness with classroom peers promote adolescents’ behavioral engagement and achievement in secondary school. J. Youth Adolesc. 46, 2341–2354. doi: 10.1007/s10964-017-0724-2, PMID: 28755252 PMC5671357

[ref63] Moreira P. A. Lee V. E. (2020). School social organization influences adolescents' cognitive engagement with school: the role of school support for learning & of autonomy support. Learn. Individ. Differ. 80:101885. doi: 10.1016/j.lindif.2020.101885

[ref64] Newcomb A. F. Bukowski W. M. Pattee L. (1993). Children’s peer relations: a meta-analytic review of popular, rejected, neglected, controversial, and average sociometric status. Psychol. Bull. 113, 99–128. doi: 10.1037/0033-2909.113.1.99, PMID: 8426876

[ref65] Ning B. T. Yang L. (2022). Analysis of the effectiveness & coordination mechanism of the policy of “reducing the burden on homework” for elementary and middle school students-based on a survey of 137 cities in 30 provinces. China Educ. Technol. 1, 9–16. doi: 10.3969/j.issn.1006-9860.2022.01.002

[ref66] Olaya M. L. González-González G. M. E. (2020). Cooperative learning projects to Foster Reading skills. GIST Educ. Learn. Res. J. 21, 119–139. doi: 10.26817/16925777.835

[ref67] Olivier E. Morin A. J. Langlois J. Tardif-Grenier K. Archambault I. (2020). Internalizing and externalizing behavior problems and student engagement in elementary and secondary school students. J. Youth Adolesc. 49, 2327–2346. doi: 10.1007/s10964-020-01295-x, PMID: 32710241

[ref68] Pan Y. (2017). The formation mechanism of college students' professional interests: the long-term impact of professional choice, social support, and academic investment. Acta Psychol. Sin. 49, 1513–1523. doi: 10.3724/SP.J.1041.2017.01513

[ref69] Putnick D. L. Bornstein M. H. (2016). Measurement invariance conventions and reporting: the state of the art & future directions for psychological research. Dev. Rev. 41, 71–90. doi: 10.1016/j.dr.2016.06.004, PMID: 27942093 PMC5145197

[ref70] Qi H. Zhang Y. Dong K. Zhao G. (2024). How dyadic emotional transmission shapes teacher-student relationship: effects of emotional convergence on cohesion in teacher-student interaction. Curr. Psychol. 43, 23469–23483. doi: 10.1007/s12144-024-06089-z

[ref71] Quin D. (2017). Longitudinal and contextual associations between teacher-student relationships and student engagement. Rev. Educ. Res. 87, 345–387. doi: 10.3102/0034654316669434, PMID: 38293548

[ref72] Reeve J. (2002). “Self-determination theory applied to educational settings” in Handbook of self-determination research. eds. Deci E. L. Ryan R. M. (Rochester, NY: University of Rochester Press), 183–203.

[ref73] Reyes M. R. Brackett M. A. Rivers S. E. White M. Salovey P. (2012). Classroom emotional climate, student engagement, andacademic achievement. J. Educ. Psychol. 104, 700–712. doi: 10.1037/a0027268

[ref74] Roorda D. L. Jak S. Zee M. Oort F. J. Koomen H. M. Y. (2017). Affective teacher-student relationships and students' engagement and achievement: a meta-analytic update & test of the mediating role of engagement. Sch. Psychol. Rev. 46, 239–261. doi: 10.17105/SPR-2017-0035.V46-3

[ref75] Ryan R. M. Deci E. L. (2020). Intrinsic & extrinsic motivation from a self-determination theory perspective: definitions, theory, practices, and future directions. Contemp. Educ. Psychol. 61:101860. doi: 10.1016/j.cedpsych.2020.101860

[ref76] Sadoughi M. Hejazi S. Y. (2022). The effect of teacher support on academic engagement: the serial mediation of learning experience and motivated learning behavior. Curr. Psychol. 42, 18858–18869. doi: 10.1007/S12144-022-03045-7, PMID: 41215982

[ref77] Sameroff A. (2009). The transactional model of development: how children and contexts shape each other. Infant Ment. Health J., 3–21. doi: 10.1037/11877-000

[ref79] Schwartz D. Gorman A. H. Nakamoto J. Toblin R. L. (2005). Victimization in the peer group and children's academic functioning. J. Educ. Psychol. 97, 425–435. doi: 10.1037/0022-0663.97.3.425

[ref80] Shin H. Chang Y. (2022). Relational support from teachers and peers matters: links with different profiles of relational support and academic engagement. J. Sch. Psychol. 92, 209–226. doi: 10.1016/j.jsp.2022.03.006, PMID: 35618371

[ref81] Sidanius J. Pratto F. (1999). Social dominance: social hierarchy and asymmetrical group behavior. New York, NY: Cambridge University Press. 9, 227–262. doi: 10.1017/CBO9781139175043.010

[ref82] Skinner E. A. Raine K. E. (2022). “Unlocking the positive synergy between engagement and motivation” in Handbook of research on student engagement. eds. Reschly A. L. Christenson S. L.. 2nd ed (Cham, Switzerland: Springer).

[ref83] Tan L. Li Q. Guo C. (2022). The impact of teacher-student relationship on learning engagement of left behind children: a moderated mediation model. Psychol. Behav. Res. 20, 782–789. doi: 10.12139/j.1672-0628.2022.06.010

[ref84] Tang D. Wen Z. (2020). Common method deviation testing: issues and suggestions. Psychol. Sci. 43, 215–223. doi: 10.16719/j.cnki.1671-6981.20200130

[ref85] Vollet J. W. Kindermann T. A. (2020). Promoting persistence: peer group influences on students' re-engagement following academic problems and setbacks. Int. J. Behav. Dev. 44, 354–364. doi: 10.1037/edu0000172

[ref86] Wang H. (2013). Research on internet addiction of left behind middle school students in rural areas & its correlation with parenting styles and peer relationships(master's thesis). Wuhan: Huazhong Normal University.

[ref87] Wang M. T. Degol J. L. Henry D. A. (2019). An integrative development-in-sociocultural-context model for children’s engagement in learning. Am. Psychol. 74, 1086–1102. doi: 10.1037/amp0000522, PMID: 31829690

[ref88] Wang M. T. Eccles J. S. (2013). School context, achievement motivation, and academic engagement: a longitudinal study of school engagement using a multidimensional perspective. Learn. Instr. 28, 12–23. doi: 10.1016/j.learninstruc.2013.04.002

[ref89] Warneken F. (2018). How children solve the two challenges of cooperation. Annu. Rev. Psychol. 69, 205–229. doi: 10.1146/annurev-psych-122216-011813, PMID: 28876999

[ref90] Wentzel K. R. Battle A. Russell S. L. Looney L. B. (2010). Social supports from teachers and peers as predictors of academic & social motivation. Contemp. Educ. Psychol. 35, 193–202. doi: 10.1016/j.cedpsych.2010.03.002

[ref92] Xu J. Yu L. Zhang X. (2024). Bridging the gender gap in academic engagement among young adults: the role of anticipated future sex discrimination and gender-role orientation. J. Youth Adolesc. 53, 2192–2201. doi: 10.1007/S10964-024-02009-3, PMID: 38755431

[ref93] Xuan X. Xue Y. Zhang C. Luo Y. H. Jiang W. Qi M. D. . (2019). Relationship among school socioeconomic status, teacher-student relationship, & middle school students’ academic achievement in China: using the multilevel mediation model. PLoS One 14:e0213783. doi: 10.1371/journal.pone.0213783, PMID: 30893361 PMC6426256

[ref95] Yang C. Bear G. G. May H. (2018). Multilevel associations between school-wide social-emotional learning approach and student engagement across elementary, middle, & high schools. Sch. Psychol. Rev. 47, 45–61. doi: 10.17105/SPR-2017-0003.V47-1

[ref96] Yang J. Yu X. Zhang J. Lu L. Yang Z. (2024). The potential category shift of primary school students' learning engagement under the background of "double reduction". Acta Psychol. Sin. 56, 295–310. doi: 10.3724/SP.J.1041.2024.00295

[ref97] Ye Z. Pang L. (1999). On the interrelationships between parent-child relationships, peer relationships, and teacher-student relationships in children. Psychol. Dev. Educ. 15, 50–53.

[ref98] Zhen R. Liu R. D. Ding Y. Jiang R. Jiang S. Hong W. (2021). Gratitude and academic engagement among primary students: examining a multiple mediating model. Curr. Psychol. 40, 2543–2551. doi: 10.1007/s12144-019-00202-3

[ref99] Zhou J. Gong X. Li X. (2024). Longitudinal relations between teacher support and academic achievement among Chinese children: disentangling between and within-student associations. J. Sch. Psychol. 103:101287. doi: 10.1016/j.jsp.2023.101287, PMID: 38432726

[ref100] Zhou Z. Sun X. Zhao D. Tian Y. Fan C. (2015). Research on the development of peer relationships. Psychol. Dev. Educ. 31:9. doi: 10.16187/j.cnki.issn1001-4918.2015.01.09

[ref101] Zhu X. Tian L. Zhou J. Huebner E. S. (2019). The developmental trajectory of behavioral school engagement & its reciprocal relations with subjective well-being in school among chinese elementary school students. Child Youth Serv. Rev. 99, 286–295. doi: 10.1016/j.childyouth.2019.01.024

[ref102] Zou H. Qu Z. Ye Y. (2007). The teacher-student relationship of primary and secondary school students & their school adaptation. Psychol. Dev. Educ. 4, 77–82. doi: 10.3969/j.issn.1001-4918.2007.04.014

[ref103] Zyphur M. J. Hamaker E. L. Tay L. Voelkle M. Preacher K. J. Zhang Z. . (2021). From data to causes iii: bayesian priors for general cross-lagged panel models (GCLM). Front. Psychol. 12:612251. doi: 10.3389/FPSYG.2021.612251, PMID: 33658961 PMC7917264

